# Magnetically Driven Elastic Microswimmers: Exploiting Hysteretic Collapse for Autonomous Propulsion and Independent Control

**DOI:** 10.1021/acsnanoscienceau.6c00014

**Published:** 2026-03-25

**Authors:** Theo Lequy, Andreas M. Menzel

**Affiliations:** † Eidgenössische Technische Hochschule Zürich, Rämistrasse 101 8092, Zürich, Switzerland; ‡ Institut für Physik, Otto-von-Guericke-Universität Magdeburg, Universitätsplatz 2 39106, Magdeburg, Germany

**Keywords:** microrobot, microswimmer, soft robotics, magnetic remote actuation, Stokes flow, targeted drug delivery

## Abstract

When swimming at low Reynolds numbers, inertial effects are negligible and reciprocal movements cannot induce net motion. Instead, symmetry breaking is necessary to achieve net propulsion. Directed swimming can be supported by magnetic fields, which simultaneously provide a versatile means of remote actuation. Thus, we analyze the motion of a straight microswimmer composed of three magnetizable beads connected by two elastic links. The swimming mechanism is based on oriented external magnetic fields that oscillate in magnitude. Through induced reversible hysteretic collapse of the two segments of the swimmer, the two pairs of beads jump into contact and separate nonreciprocally. Due to higher-order hydrodynamic interactions, net displacement results after each cycle. Different microswimmers can be tuned to different driving amplitudes and frequencies, allowing for simultaneous independent control by just one external magnetic field. The swimmer geometry and magnetic field shape are optimized for maximum swimming speed using an evolutionary optimization strategy. Thanks to the simple working principle, an experimental realization of such a microrobot seems feasible and may open new approaches for microinvasive medical interventions such as targeted drug delivery.

## Graphical abstract

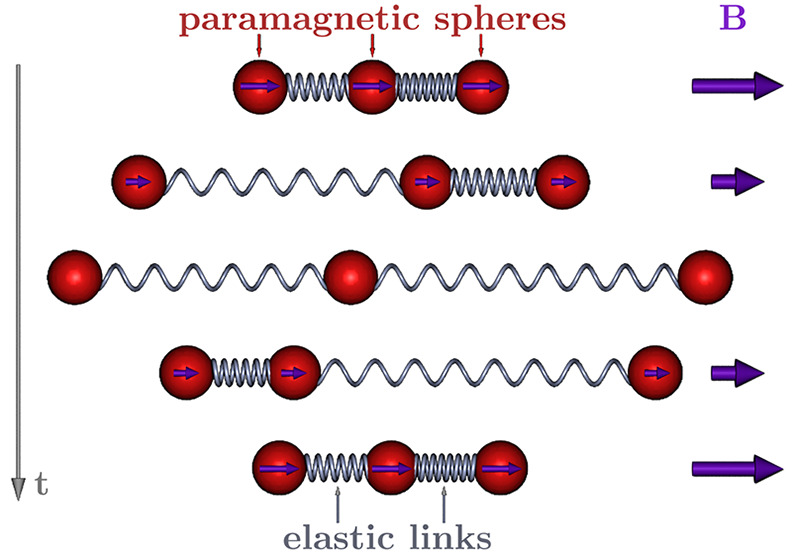

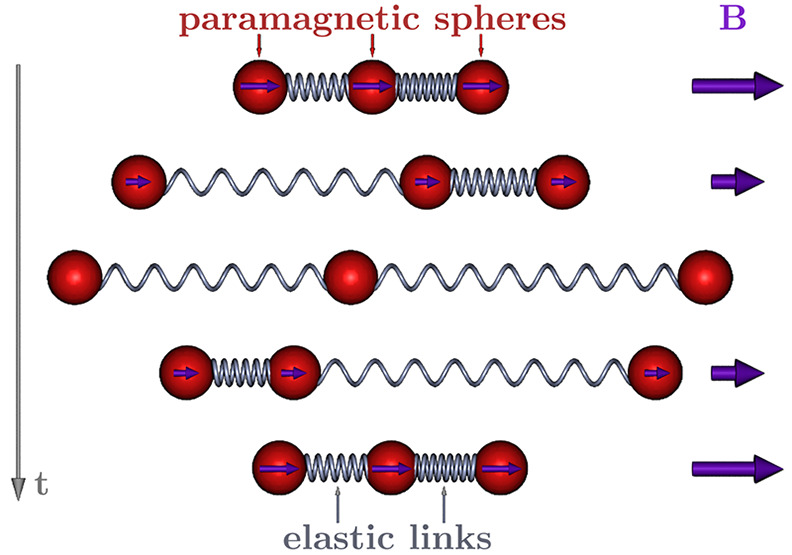

## 1 Introduction

The field of microrobotics promises significant advances in biomedicine, because it allows microinvasive interventions, such as targeted drug or cell delivery.^1,2^ Such microscopic robots could further be used for micromanipulation and targeted removal of toxic substances in the environment.^3^ Despite the benefits of the small length scales of microrobots on the order of micrometers, these scales also impose restrictions on design and mode of operation.

Here, we mostly address the central challenges of actuation and swimming at low Reynolds numbers along a requested direction. As it becomes very difficult to equip such small robots with on-board power devices, and since potential fuel usually cannot be distributed in their environment in medical applications, most microrobots resort to remote actuation. Besides visible light^4^ and ultrasound,^5^ magnetic fields are a popular choice.^6^ They can induce magnetic moments in magnetizable materials and exert torques by aligning them with the direction of the magnetic field. Moreover, net forces can be achieved through magnetic field gradients. Yet, this external actuation, at first glance, poses challenges on the construction of a microrobot. All magnetizable components of the microswimmer (in our case three magnetizable beads) are addressed simultaneously by the external field. So how can we achieve control over the different internal degrees of freedom to achieve required nonsimultaneous patterns of motion, although they share the same actuation channel via the magnetic field? Moreover, it appears difficult to steer individual microswimmers separately when multiple of them are present in one external magnetic field.

Generally, swimming at low Reynolds numbers differs from our daily experience at macroscopic scales. In Stokes flow at small length scales, viscous forces dominate and fluid inertia is negligible, causing flows to be time-reversible. A sequence of shape changes that is identical when played forward and backward, known as reciprocal motion, therefore does not generate time asymmetry, even if performed at different speed. As a result, the net displacement after a full reciprocal cycle is zero.^7^ Effective propulsion in such environments requires nonreciprocal deformation that breaks time-reversal symmetry. One single linear degree of freedom is insufficient, because any cyclical motion is reciprocal in that case.

Designing microswimmers is thus an act of balance between simplicity, to facilitate fabrication and operation, and complexity, to achieve nonreciprocal motion or otherwise break forward–backward symmetry. Nature has evolved various strategies to achieve this, including rotating helical flagella, snake-like undulations, or shape morphing.^8^ Some of them can be mimicked in artificial microswimmers.^9–12^

A minimal model microswimmer that can achieve net propulsion at low Reynolds numbers is the three-sphere swimmer introduced by Najafi and Golestanian.^13^ It consists of three collinearly arranged spheres connected by two arms of variable length. By changing the lengths of the two arms in a nonreciprocal manner, net motion is achieved. This model has been studied extensively.^13–20^

Although this linear arrangement lends itself to analytical treatment and is easy to visualize, its practical realization remains a challenge. Previous experimental attempts have involved optical tweezers to replicate the movement pattern,^15^ which is however impractical for in vivo applications. The implementation of a self-assembled microswimmer has been achieved relying on capillary forces at fluid interfaces combined with periodic magnetic actuation.^21^ Yet, it seems that involving the role of fluid surfaces may limit applications inside biological systems. Symmetry breaking was achieved by using spheres of different sizes while tuning the actuation frequency accordingly, and the listed model equations involve inertial effects.

In the following, we propose a different approach, still based on magnetic actuation. In our analysis, we connect the three magnetizable spheres using two elastic springs. We entirely concentrate on the overdamped regime, which generally renders nonreciprocality a challenge. To succeed, we focus on an effect investigated earlier in theory and experiments. It refers to the reversible collapse and reseparation of pairs of elastically linked beads, which can be induced magnetically.^22–26^ The hysteretic nature of this phenomenon allows the collapse and reseparation to occur at different field strengths, thus breaking time-reversal symmetry.

First, we analytically describe the hysteretic behavior of the collapse of two spheres linked by a finitely extensible spring in Section 2.1. Then, we introduce the complete three-sphere swimmer and find conditions for nonreciprocal motion, see Section 2.2. We proceed by analyzing hydrodynamic interactions in Section 2.3 and provide a geometric illustration for how the loop in the configuration space leads to net propulsion. Using an evolutionary strategy algorithm, we optimize the design of the microrobot and the driving magnetic field to achieve maximum speed in Section 2.4. Finally, we conclude in Section 3 with brief ideas on potential implementations and improvements in design.

## 2 Results and Discussion

### 2.1 Hysteretic Reversible Magnetically Induced Collapse

We start by an analytical consideration of the hysteretic collapse and reseparation of two magnetized spheres linked by a finitely extensible spring shown in Figure 1.

**Figure 1:**
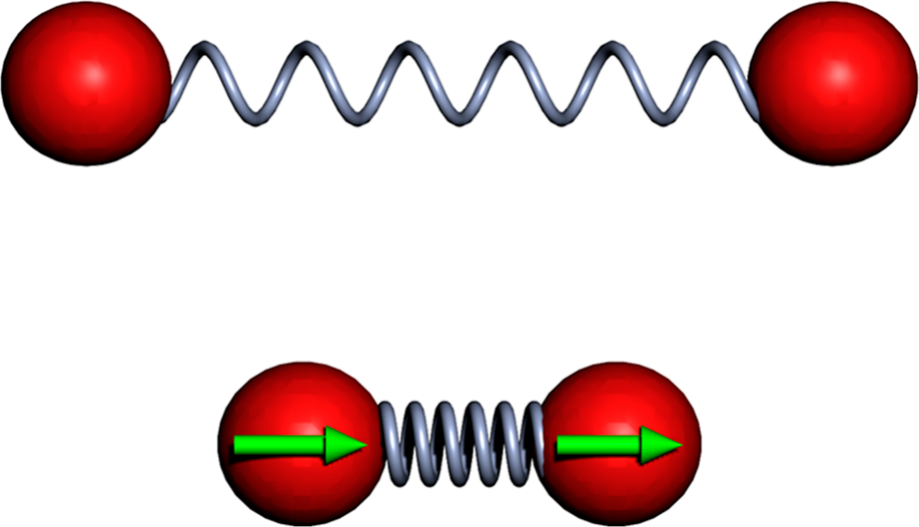
Two magnetic spheres (red) linked by a finitely extensible spring (silver-blue). The top snapshot shows the nonmagnetized configuration with the spring in its undeformed state. In the bottom constellation, a magnetic field is applied along the axis connecting the centers of the two spheres. It induces the magnetic moments *m* (green arrows) in the spheres, leading to an attractive magnetic force, which contracts the spring. Due to the finite extensibility of the spring, the spheres cannot be brought arbitrarily close together.

The spheres of radius *a* consist of a superparamagnetic material with high magnetic susceptibility χ ≫ 1. When applying a homogeneous external magnetic field *H*=H*x̂* in the *x*-direction, each sphere develops a magnetic dipole moment *m* = χ_*a*_ *VH*, where *V* = 4π*a* ^3^/3 is the volume of the sphere. We hereby neglect mutual magnetizing effects among the spheres. That is, we assume that their separations are sufficiently large, and/or the external magnetic field is sufficiently strong to magnetize the spheres toward saturation. The apparent susceptibility χ_*a*_ = χ/(1 + *N*χ) is reduced due to demagnetization, with a demagnetizing factor *N* = 1/3 for spheres. Thus, the attractive dipole–dipole interaction between the two spheres can be described by the potential

1 $$U_{mag}=-\frac{8\pi }{9}\chi_{a}^{2}\mu_{0}a^{6}\frac{H^{2}}{x^{3}}$$

where *x* is the center-to-center distance between the spheres and μ_0_ is the vacuum permeability. To counterbalance this attraction, the spheres are connected by a finitely extensible nonlinear elastic spring (FENE)^27^ of rest length l in the undeformed state, maximum change in length l _max_ under extension or compression, and stiffness *k*, with the spring potential

2 $$U_{FENE}=-\frac{1}{2}kl_{\max}^{2}\;\ln\left[1-\frac{{\left(x-l\right)}^{2}}{l_{\max}^{2}}\right]$$

Dimensional analysis shows that the relevant dimensionless parameters are the extensibility ϵ = l _max_/ l of the spring and the rescaled magnetic field strength $\beta =H\sqrt{8\pi \chi_{a}^{2}\mu_{0}a^{6}/3kl^{5}}$ . All lengths can be scaled by l and energies by *k* l ^2^. The total rescaled potential for two spheres as a function of the relative separation *r* = *x*/ l then reads

3 $$U_{2}=-\frac{\epsilon^{2}}{2}\ln\left(1-\frac{{\left(r-1\right)}^{2}}{\epsilon^{2}}\right)-\frac{1}{3}\frac{\beta^{2}}{r^{3}}$$

For small values of β, the spring potential dominates, and there is a single stable equilibrium position close to the rest length l . While increasing β, a second local minimum appears in a saddle-node bifurcation at small separations due to magnetic attraction. When the field strength is increased further, the first minimum disappears in another saddle-node bifurcation, and only the collapsed state remains stable. This behavior is illustrated in Figure 2.

**Figure 2:**
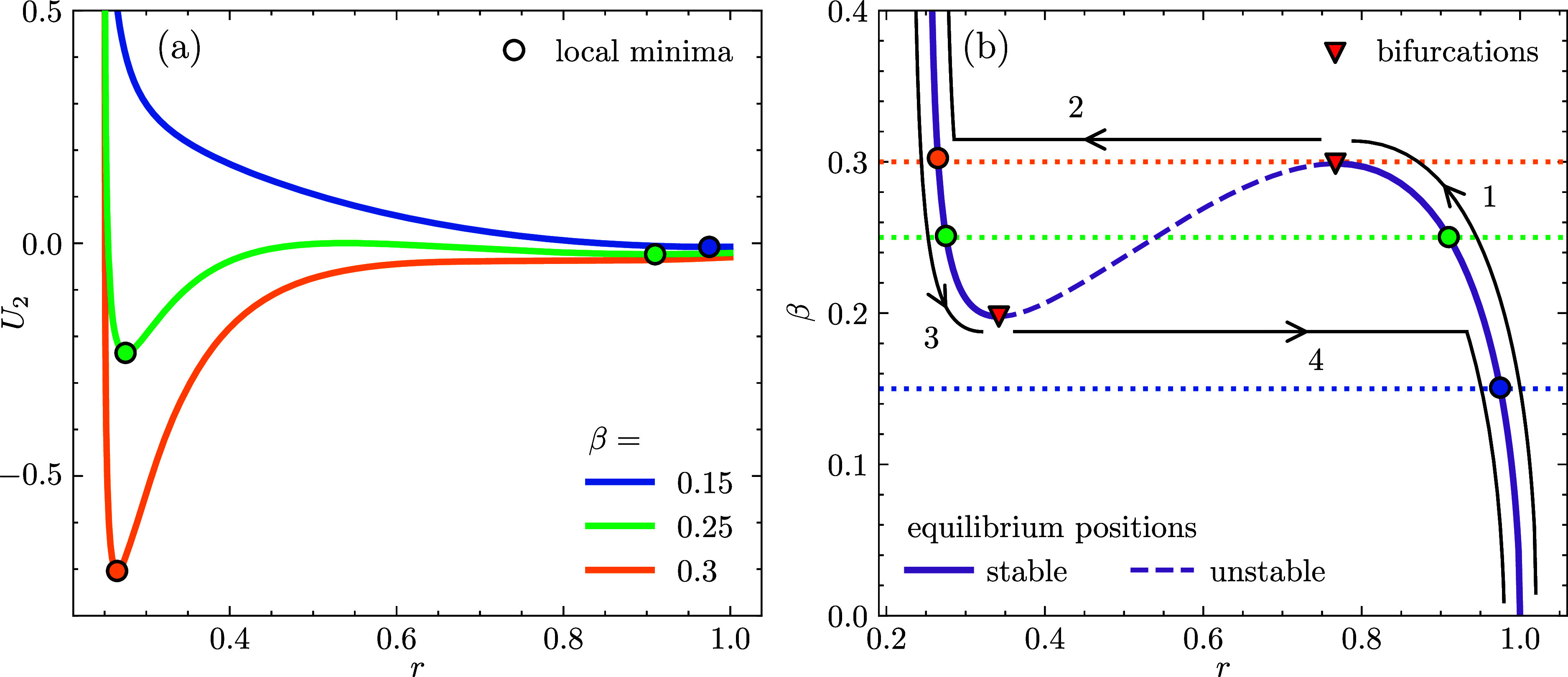
(a) Two magnetizable spheres are subject to the dimensionless potential *U*_2_, see eq 3, when linked by a FENE spring and exposed to an external magnetic field of rescaled strength β. *r* denotes the rescaled center-to-center distance between the spheres. The extensibility of the spring is set to ϵ = 0.75. When the magnetic field strength β is varied, different local minima (circles) appear. For β = 0.15 (blue), there is only one stable configuration close to the rest length of the spring, *r* ≈ 1. When increasing β to 0.25 (green), the minimum moves slightly to the left and a second minimum appears in a compressed state of the spring, *r* ≈ 0.25, due to the induced magnetic attraction. For β = 0.3 (orange), the magnetic attraction dominates, leading to the disappearance of the minimum on the right so that the compressed state is the only stable minimum. (b) Bifurcation diagram for this configuration in the plane spanned by the rescaled length of the spring *r* and the rescaled magnitude of the external magnetic field β, see eq S1. (1) When the magnetic field is increased, the stable equilibrium position (solid purple line) moves to smaller lengths *r* of the spring, until the solution vanishes in a saddle-node bifurcation (red triangle). (2) From there, the spheres collapse to a compressed state of the spring. (3) When the field is decreased again, the spheres remain in the collapsed state for magnetic field amplitudes β lower than those of the previous event of collapse, implying hysteresis. (4) Finally, another saddle-node bifurcation appears, and the spheres abruptly detach and reseparate. At intermediate field strengths β, between both saddle-node bifurcations, two stable equilibria coexist, separated by an unstable solution (dashed purple line). The three different field strengths β in (a) are marked by horizontal, dotted, colored lines. Corresponding minima in the potential in (a) are indicated by circled, colored dots.

The points of bifurcation depend only on the extensibility ϵ of the spring and are given as the roots of a cubic polynomial, see part A of the Supporting Information. Bifurcations only appear above a critical extensibility $\epsilon =1/\sqrt{3}$ . With increasing extensibility ϵ, the separation between the two branches resulting from the bifurcation increases, see Figure 3a,b. This concerns both separation in space *r*, see Figure 3a, and associated field strengths β, see Figure 3b. In Figure 3b, the required field strength for the collapse (blue dash-dotted line) does not vary significantly with changes in extensibility. It mainly depends on the initial spring stiffness. However, as the spheres get closer, with increasing extensibility/compressibility of the springs, the magnetic field has to be lowered more until the spheres detach again (green dashed line). Individual bifurcation diagrams for different extensibilities ϵ are illustrated in Figure 3c.

**Figure 3:**
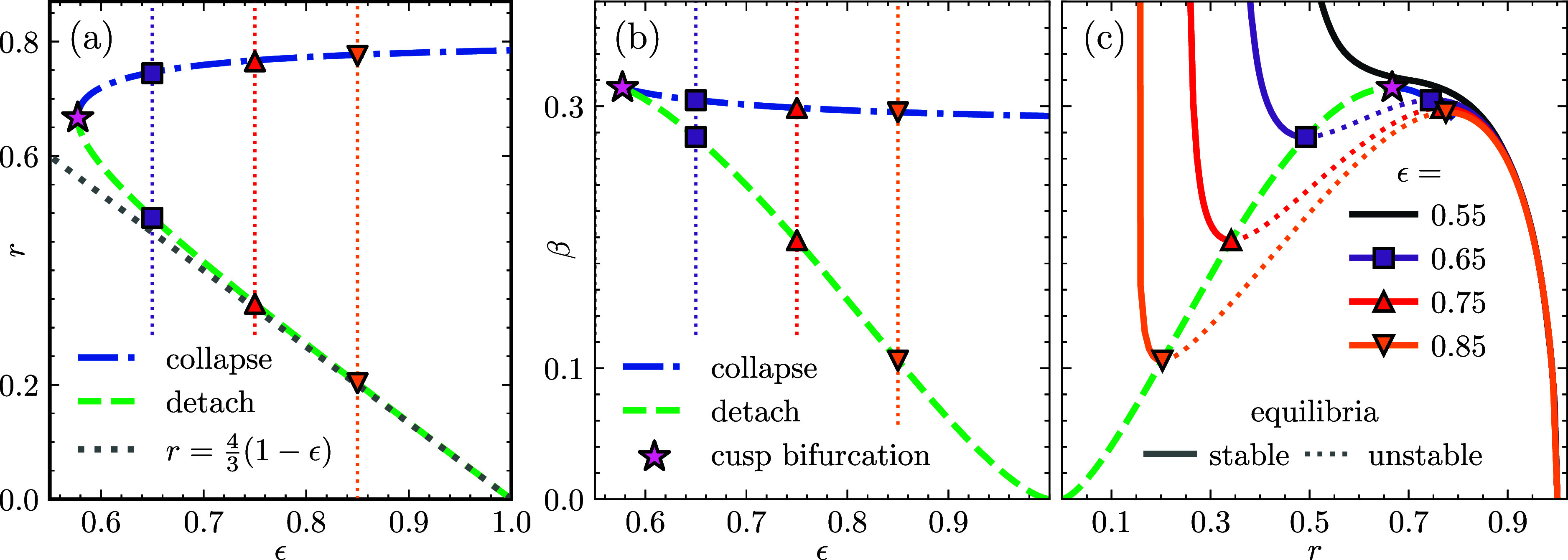
(a) Location of the bifurcations depending on the extensibility ϵ = l _max_/ l . With increasing extensibility, the two bifurcation values of the distance *r* between the centers of the spheres corresponding to collapse (blue dash-dotted line) and detaching (green dashed line) further separate. The detaching position has the asymptotic behavior of *r* = *x*/ l = 4­(1 – ϵ)/3 (gray dotted line) when the extensibility approaches unity. At $\epsilon =1/\sqrt{3}\approx 0.6$ , both curves corresponding to saddle-node bifurcations merge into a cusp bifurcation (rose star). Below this critical value of the extensibility, no bifurcations occur. In (b) the magnetic field strengths β, at which the bifurcations occur, are shown for different extensibilities. The individual bifurcation diagrams for the marked points are shown in (c), together with the curve traced out by all points of bifurcation (green dashed line). The values of the extensibilities ϵ = 0.65, 0.75, and 0.85 for the depicted bifurcation scenarios (purple, red, and yellow, respectively) in (c) are marked in (a) and (b) for reference as vertical dotted lines of identical color.

### 2.2 Nonreciprocal Motion of the Three-Sphere Microswimmer

We now introduce the full three-sphere microswimmer consisting of three magnetizable beads with radius *a*, connected by FENE springs of rest lengths l _*i*_, maximum extensions l _*i*,max_, and stiffnesses *k*_*i*_, where *i* ∈ {1, 2}. We assume the springs to be resistant to bending and torsion so that the spheres remain collinearly aligned. The mutual dipolar interaction together with the aligning effect of the external magnetic field support this collinear alignment, in particular during potential fabrication using methods of self-assembly.^28–30^ If the external magnetic field is applied along the *x*-direction, the swimmer aligns itself with the field because its overall easy axis of magnetization is along its long axis. Hence, we only consider motion along the *x*-axis in the following.

The first spring connects the first two spheres at center locations *x*_1_ and *x*_2_, while the second spring connects the second to the third sphere at center locations *x*_2_ and *x*_3_. We denote the center-to-center distances as *x*_*ij*_ = *x*_*j*_ – *x*_*i*_, where *i* = 1 and *j* = 2 or *i* = 2 and *j* = 3. Next, we rescale all lengths by the radius *a* and the magnetic field as *h* = *H*/*H*_max_, where *H*_max_ denotes the maximally attainable magnetic field strength. To simplify the numeric prefactors, we rescale energies by 8πχ_*a*_^2^μ_0_ *a* ^3^ *H*_max_^2^/3 and the spring constants by 8πχ_*a*_^2^μ_0_ *aH*_max_^2^/3.

In total, the potential of our three-sphere swimmer is then given by

4 $$\begin{array}{lll} U_{3} & = & -\frac{1}{2}k_{1}l_{1,\max}^{2}\;\ln\left(1-\frac{{\left(x_{12}-l_{1}\right)}^{2}}{l_{1,\max}^{2}}\right)\\ & & -\frac{1}{2}k_{2}l_{2,\max}^{2}\;\ln\left(1-\frac{{\left(x_{23}-l_{2}\right)}^{2}}{l_{2,\max}^{2}}\right)\\ & & -\frac{h^{2}}{3}\left(\frac{1}{x_{12}^{3}}+\frac{1}{x_{23}^{3}}+\frac{1}{{\left(x_{12}+x_{23}\right)}^{3}}\right), \end{array}$$

where all quantities are now dimensionless. The dynamics of *x*_12_ and *x*_23_ are only loosely connected, because the magnetic coupling term $-h^{2}/\left[3{\left(x_{12}+x_{23}\right)}^{3}\right]$ is comparatively small due to the larger distance between the outer spheres. However, the presence of the additional sphere tends to reduce the equilibrium distance between each pair of spheres in a magnetized state and lowers the required field strengths for collapse and detaching. The effect is stronger when the other outer sphere is already in the collapsed state. Hence, the collapse of one spring can trigger the collapse of the other, and similarly for the expansion events. Moreover, abrupt movements of the center sphere during bifurcation events affect the spring lengths of both linked pairs of spheres. Corresponding effects are included in the following analysis of the three-sphere swimmer, while the underlying driving hysteretic effect remains the one described in Section 2.1.

We now apply a slowly oscillating magnetic field *h*(*t*) that varies between a minimum magnitude *h*_min_ and a maximum magnitude *h*_max_ = 1, without changing direction. During one cycle of the magnetic field, both pairs of spheres will undergo hysteretic collapse and detachment at different field strengths. The desired order of those events to achieve nonreciprocal motion and thus net propulsion of our microrobot is as follows. Starting from high magnetic field strengths in the collapsed state, when gradually decreasing the magnetic field, one pair of spheres separates first. Then the other pair of spheres separates. This secondary event is supported by the now weaker magnetic attraction toward the already separated outer sphere from the first separation event. When the strength of the magnetic field is increased again, the sequence of reaction is the same for the two pairs. The pair that first separated will also be the first to collapse. Then the other pair follows. The cycle is completed, and the swimmer is again in its initial state.

We denote the necessary rescaled magnetic fields for separation and collapse of the pair of spheres *i* and *j* as *h*_separate,*ij*_ and *h*_collapse,*ij*_, respectively. For our purpose, we require *h*_separate,23_ < *h*_separate,12_ ≤ *h*_collapse,12_ < *h*_collapse,23_ ≤ 1. It appears from here that the extensibility of the spring length *x*_23_ should be larger. This promotes a wider gap between the field strengths of separation and collapse. In principle, it is not necessary for the spring length *x*_12_ to show any hysteretic behavior at all, as long as the major deformation of this spring occurs between *h*_separate,23_ and *h*_collapse,23_. For the magnetic field to be able to induce the full collapse, we require *h*_collapse,*ij*_ ≲ 1, which implies $k_{i}l_{i}^{5}≲10$ , see part B of the Supporting Information. We therefore choose to define the stiffness parameters $c_{i}=k_{i}l_{i}^{5}$ for *i* ∈ {1, 2}. Since these stiffness parameters are related to the strength of the magnetic field at collapse, they form a reasonable choice of parametrization adapted to the investigated situation. We found them more suitable than the pure spring constants *k*_*i*_ when performing the optimization below.

### 2.3 Hydrodynamic Interactions and Net Propulsion

At low Reynolds numbers, the motion of the microswimmer is overdamped and governed by Stokesian hydrodynamics. Since the flow equations are linear, they lead to equations of motion for the positions of the spheres of the form

5 $$v_{i}=\frac{dx_{i}}{dt}=M_{0}\sum_{j=1}^{3}M_{ij}F_{j},,i=1,2,3$$

here, *F*_*j*_ = – ∂*U*_3_/∂*x*_*j*_ are the forces that act on the spheres due to the potential in eq 4. *M*_*ij*_ are the components of the dimensionless mobility matrix *M*. The latter was rescaled by the mobility of a single sphere *M*_0_ = 1/6πη*a*, where η is the shear viscosity of the incompressible fluid. If bending becomes significant in reality during magnetic actuation, for example, if the elastic links are substantially softer under large-scale bending than under compression, against the stabilizing collinear action of the magnetic field, or if the direction of the magnetic field is changed, eq 5 must be evaluated in three dimensions. In that case, rotational degrees of freedom must be included and the three-dimensional mobility matrix including translation–rotation couplings must be evaluated.^18^ At present, we focus on the persistently collinear case that can be reduced to one dimension. To render this equation dimensionless and to simplify the prefactor, we rescale time by the characteristic time scale 9 × 10^6^η/4χ_*a*_^2^μ_0_ *H*_max_^2^ and velocities accordingly by 4 × 10^–6^χ_*a*_^2^μ_0_ *H*_max_^2^ *a*/9η. In these units, the mobility of a single sphere becomes *M*_0_ = 10^6^.

With the help of Faxén’s relations, the mobility matrix can be expanded in terms of the inverse intersphere distances 1/*x*_*ij*_ as given in part C of the Supporting Information. The expansion breaks down when the spheres are very close to each other. To avoid this regime, we impose a minimum separation of 4*a* between the centers of the spheres. For this purpose, we restrict the maximum deviation from the rest length of the springs by setting l _*i*,max_ = l _*i*_ – 4, so that the FENE potential in eq 2 diverges before the distance between the spheres becomes too small. Furthermore, we consider the springs as frictionless. This assumption simplifies the numerical treatment. Yet, experimental results will deviate to a certain degree, depending on the exact realization of the springs. In general, through additional dampening the dynamics becomes slower such that either the driving frequency has to be reduced or the magnetic field strength and spring stiffness have to be increased to compensate for this drag. Furthermore, hydrodynamic interactions with and between the springs affect the net per-cycle displacement. To some extent, hydrodynamic friction of spring segments may be combined with that of the nearby sphere in the calculation, yet it changes between the compressed and expanded state.

Purcell’s scallop theorem^7^ in our case implies that the net propulsion of the swimmer after one cycle of the magnetic field only depends on the shape of the loop in configuration space (*x*_12_, *x*_23_). We now provide a geometric interpretation of this cycle. For this purpose, we change to relative coordinates by transforming

6 $$x=\left(\begin{array}{l} x_{1}\\ x_{2}\\ x_{3} \end{array}\right)\to \underset{≕U}{\underset{︸}{\left(\begin{array}{lll} -1 & 1 & 0\\ 0 & -1 & 1\\ 0 & 1 & 0 \end{array}\right)}}\cdot \left(\begin{array}{l} x_{1}\\ x_{2}\\ x_{3} \end{array}\right)=\left(\begin{array}{l} x_{12}\\ x_{23}\\ x_{2} \end{array}\right)=x_{rel}$$

Introducing the vector *e* = (1,1,1)^*T*^ to project onto the total force, Newton’s third law $\sum_{i=1}^{3}F_{i}≕e^{T}\cdot F=0$ can be rewritten as

7 $$0=\underset{≕\alpha^{T}}{\underset{︸}{e^{T}\cdot M^{-1}\cdot U^{-1}\;}}dx_{rel}=\alpha_{1}dx_{12}+\alpha_{2}dx_{23}+\alpha_{3}dx_{2}$$

Now we move into the configuration space (*x*_12_, *x*_23_). Since the total displacement does not integrate to a function of the configuration space, we denote it as an inexact one-form *đx*_2_.

8 $$đx_{2}=-\frac{\alpha_{1}}{\alpha_{3}}dx_{12}-\frac{\alpha_{2}}{\alpha_{3}}dx_{23}$$

The displacement over one cycle is then given by the line integral over the configuration space loop γ

9 $$\Delta x_{2}=\oint_{\gamma }đx_{2}=\int_{S\left(\gamma \right)}dđx_{2}≕\int_{S\left(\gamma \right)}F$$

where we have used Stokes theorem to convert the line integral into a surface integral over the area *S*(γ) enclosed by the loop γ. We here defined the two-form $F=dđx_{2}=fdx_{12}\wedge dx_{23}$ , together with

10 $$f=\left(-\frac{\partial }{\partial x_{12}}\left(\frac{\alpha_{2}}{\alpha_{3}}\right)+\frac{\partial }{\partial x_{23}}\left(\frac{\alpha_{1}}{\alpha_{3}}\right)\right)$$

*f* is the infinitesimal displacement per area in configuration space. Introducing a configuration space metric *g* corresponding to the dissipated energy, the scalar $f/\sqrt{\det g}$ can also be interpreted as the curvature of the configuration space.^31^

As can be seen in the phase space Figure 4a, there are three distinct dynamical states. In the state that achieves the highest net displacement (red diamond), the collapses occur in the correct sequence described above, leading to a large loop in configuration space, see Figure 4b. To achieve this extended loop, it is essential that the springs notably differ both in rest length and in stiffness. If, from there, in Figure 4a the stiffness ratio *c*_1_/*c*_2_ is decreased (orange circle), the detachment of the first pair of spheres occurs after the separation of the second pair. The enclosed area in configuration space is much smaller, and the net displacement decreases significantly. If, instead, the stiffness ratio *c*_1_/*c*_2_ is kept constant and the rest length ratio l _1_/ l _2_ is decreased (pink triangle), the collapse of the second pair occurs earlier than that of the first pair. The consequence is again a much smaller loop in configuration space, together with a significantly lower, sometimes even negative, net displacement. Between the circle and the diamond, there is a relatively sharp transition between the different dynamic states. The transition between these states apparently vanishes at a critical point when *c*_1_/*c*_2_ ≈ 0.75 and l _1_/ l _2_ ≈ 0.4.

**Figure 4:**
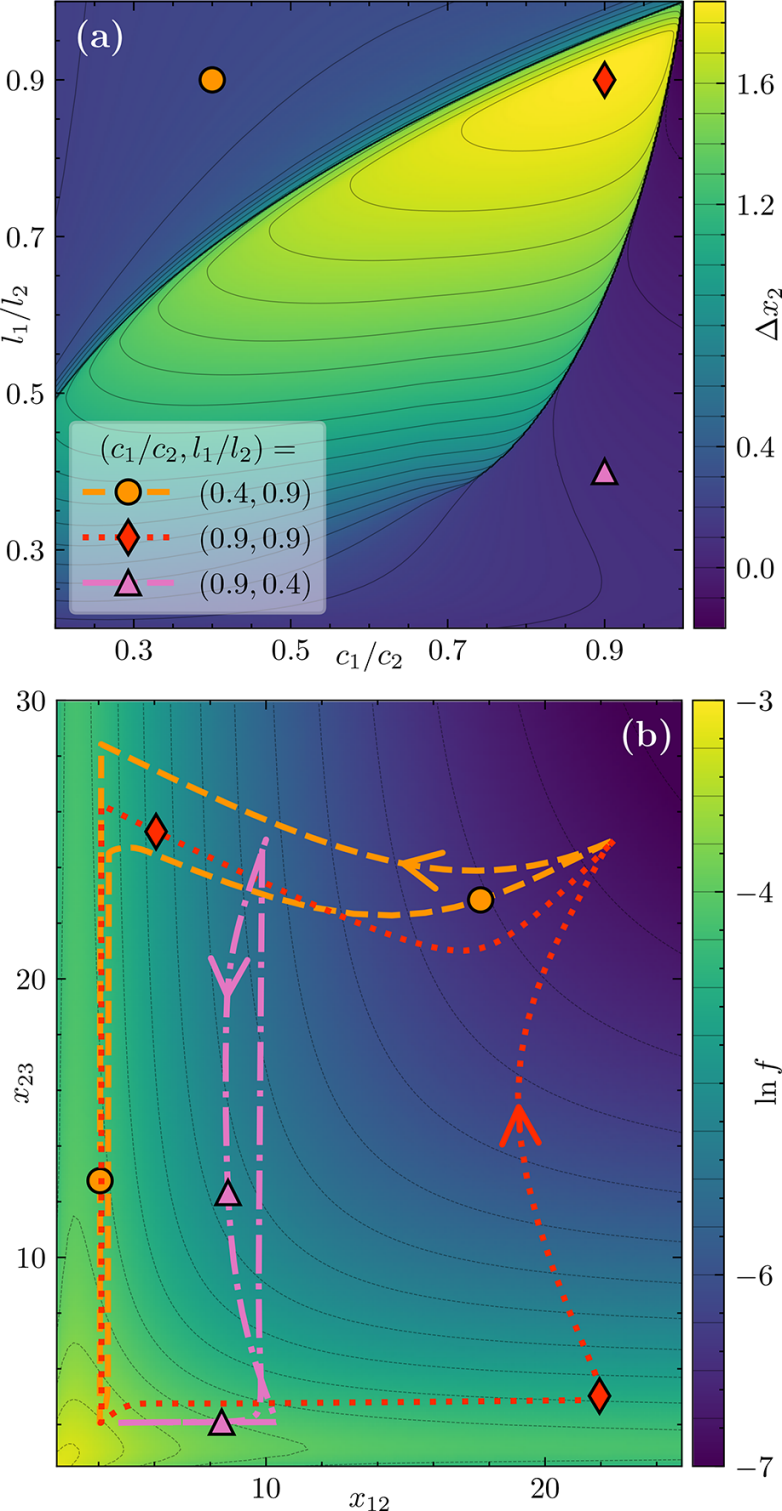
(a) Color map of the net displacement Δ*x*_2_ after one full cycle of the magnetic field for swimmers with different ratios of spring stiffness *c*_1_/*c*_2_ and rest lengths l _1_/ l _2_ (phase space). For all swimmers *c*_2_ = 10, l _2_ = 25, and in the fully compressed state the center-to-center distance is still larger than l _*i*_ – l _*i*,max_ = 4. The magnetic field is ramped up from 0 to 1 with the very slow rate d*h*/d*t* = 10^–3^ and then back down again to 0. Except for the points of bifurcation, the swimmer is almost always in equilibrium. The three markers each label one swimmer that represents one of the three dynamical states in this phase space. Their trajectories in configuration space are shown in (b). (b) Color map of the natural logarithm of the infinitesimal displacement *f* per area in configuration space (*x*_12_, *x*_23_) calculated according to eq 10 from the sixth-order expansion of the mobility matrix eq S8. Three loops in configuration space traced out during one cycle of the magnetic field by the three-sphere swimmers with the different parameters as marked in (a).

For smaller length ratios, the length of the first spring *l*_1_ < 10 is too short, and the extensibility is below ϵ_1_ = l _1,max_/ l _1_ < 0.6. As shown in Section 2.1 for two spheres, no bifurcations, and thus no hysteretic behavior occurs for such low extensibilities. Due to its absence, the transition is thus more continuous.

### 2.4 Evolutionary Optimization of Swimmer Design and Driving Field

The design of the swimmer can be optimized to achieve maximum swimming speed under practical constraints, see Methods. To ensure real-world applicability, we now choose dimensional parameters. The spheres of radius *a* = 1 μm are made of a superparamagnetic material with high magnetic susceptibility χ ≫ 1, suspended at room temperature in water with dynamic viscosity η = 7 × 10^–4^ Pa s and density ρ = 1000 kg/m^3^. Generally, higher external magnetic fields lead to stronger induced magnetic forces and thus larger speeds of the spheres, relating to the overall speed of the swimmer. Simultaneously, experimental realizability and required safety of in vivo applications impose constraints on the field strength. Hence, for the following numeric study, the magnetic field is restricted in magnitude by *H*_max_ = 1× 10^3^ Oe = 100 mT/μ_0_ and can be varied at a maximum rate of |d*H*/d*t*| ≤ 2.7 × 10^4^ Oe/s following the ICNIRP guidelines^32^ to remain safe for in vivo applications. Thus, using the dimensionless *h* and *t* as introduced in Sections 2.2 and 2.3, |d*h*/d*t*| ≤ 0.59. A simple driving magnetic field takes the form of a cosine wave that oscillates between *h*_min_ and *h*_max_ with angular frequency ω. In this case, the constraints on the field magnitudes translate to |*h*_max_| ≤ 1, |*h*_min_| ≤ 1, and ω|*h*_max_ – *h*_min_|/2 ≤ 0.59. For the swimmer, we restrict the length l _*i*_ – l _*i*,max_ ≥ 4 to guarantee that the surfaces of the spheres always remain at least one diameter apart. Thus, we avoid lubrication effects and reduce mutual magnetization. Moreover, we choose the spring stiffness $c_{i}=k_{i}l_{i}^{5}$ in the range 1 ≤ *c*_*i*_ ≤ 20, and we set the rest lengths 5 ≤ l _*i*_ ≤ 50.

Even though gradients are available through automatic differentiation, gradient-based optimization approaches were not successful. This is because the optimization landscape is highly nonconvex and contains sharp cliffs at the transitions between different dynamics states, which can be seen in Figure 4a. We thus optimize this 7-dimensional problem over ( l _1_, l _2_, *c*_1_, *c*_2_, *h*_min_, *h*_max_, ω) using the covariance matrix adaptation evolutionary strategy (CMA ES)^33^ with a population size of 32 for 150 generations, until the solution stops improving. The final solution is shown in Figure 5. In physical units, this swimmer of total outer length on the order of 33 μm swims at an average speed of 18.6 μm s^–1^ when driven by a magnetic field oscillating between 520 and 884 Oe at a frequency of 23.5 Hz. The spring stiffnesses resulting from the optimization process are *k*_1_ = 1.2 × 10^–4^ N/m and *k*_2_ = 4.1 × 10^–5^ N/m.

**Figure 5:**
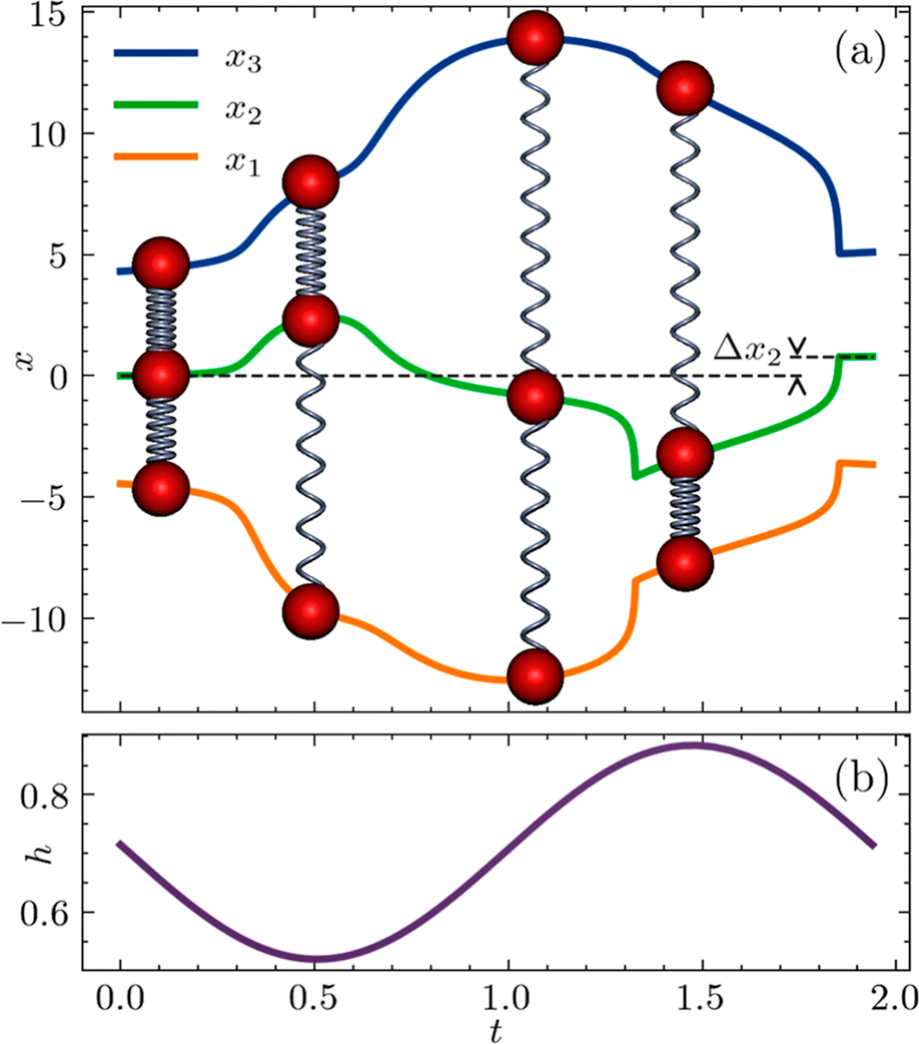
(a) One cycle of the trajectories of a three-sphere microswimmer with optimized parameters *c*_1_ = 6.63, *c*_2_ = 8.22, l _1_ = 13.7, l _2_ = 17.0 and l _*i*,max_ = l _*i*_ – 4. The net displacement accrued over one cycle of the magnetic field is Δ*x*_2_ = 0.791 in dimensionless units. This gives an average speed of *v* = 0.408 over the full cycle or *v* = 18.6 μms^–1^ in physical units when using the scales described in Section 2.4. In each of the four stages of the cycle, a snapshot of the swimmer consisting of three spheres (red) and two springs (silver-blue) is shown, where the spheres are depicted to scale. (b) The driving magnetic field *h*(*t*) oscillates between *h*_min_ = 0.52 and *h*_max_ = 0.884 with angular frequency ω = 3.24.

Due to the constraints imposed on the slew rate of the magnetic field d*h*/d*t*, the optimized driving field does not drop to zero but stays in the interval where hysteretic effects are important. This, in turn, allows for the frequency to be increased, which improves the speed. Besides, the optimized driving field never reaches the maximum allowed strength (1 in rescaled units). Thus, using stronger electromagnets while maintaining the restriction on the slew rate would not further enhance the overall swimming speed.

Since the driving frequency is much faster than in the quasistatic case described in Section 2.3, the detaching and collapse do not happen instantaneously, but with significant delay. The swimmer reaches its fully collapsed state only after the magnetic field has reached maximum amplitude and is already declining again. During this cycle, the maximum speed of a single sphere *v*_max_ = 1.97 × 10^–2^ m/s is reached during the collapse of the first spring at *t* = 1.72. Thus, the Reynolds number is

11 $$Re=\frac{2\rho v_{\max}a}{\eta }=5.7\times 10^{-2}\ll 1$$

so that the assumption of Stokes flow still remains approximately valid.

If we allow for more general shapes of the forcing magnetic field *h*(*t*), the velocity can be further increased. The term *h* ^2^(*t*) enters affinely into the total force, resulting from the potential in eq 4. Consequently, by Pontryagin’s maximum principle^34^ from optimal control theory, the magnetic field which leads to the extremal velocity must lie on the boundary of the manifold, that is, be a bang–bang control. Thus, at each point in time *t*, either *h*(*t*) ∈ {0, 1} or d*h*/d*t* = ± 0.59 locates extrema, according to the bounds of optimization introduced above. Therefore, a reasonable class of driving fields is given by triangle wave functions clipped to the interval [0,1]. Performing the same evolutionary optimization strategy within this class gives rise to a swimmer whose cycles are shown in Figure 6. The amplitude of the magnetic field in Figure 6b remains similar to the one of the cosine-shaped field in Figure 5b. Yet, the frequency can be increased while keeping similar displacement per period and thus the swimmer is around 18% faster.

**Figure 6:**
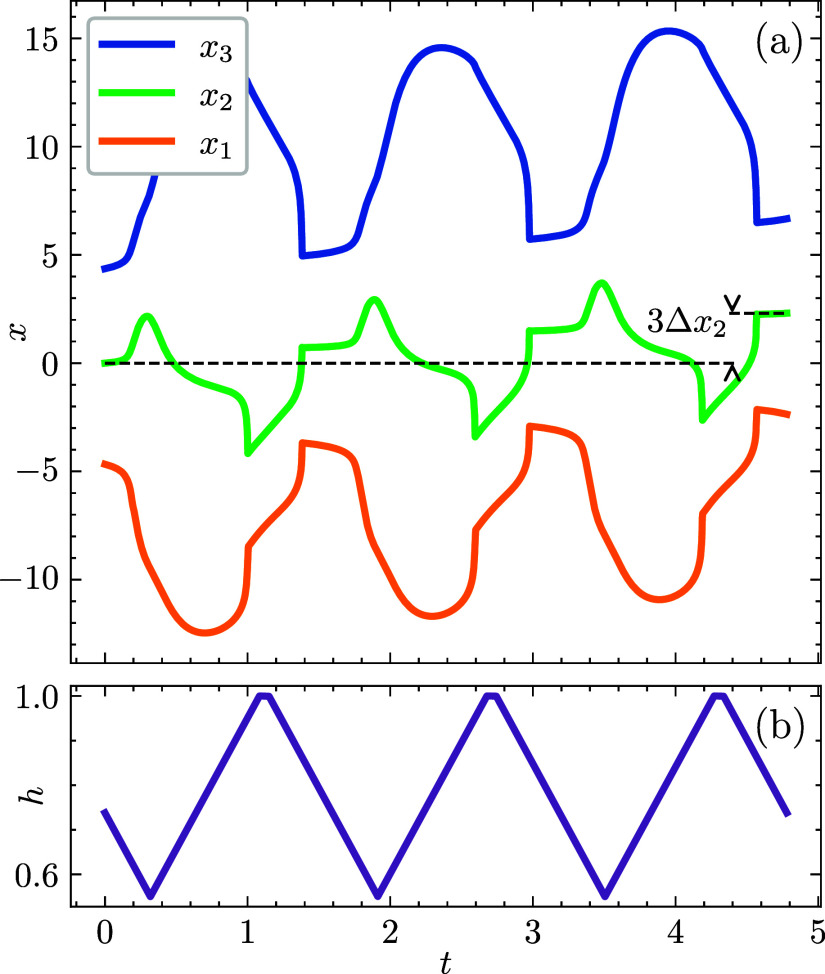
(a) Three cycles of the trajectories of a three-sphere microswimmer with parameters *c*_1_ = 8.1, *c*_2_ = 9.8, l _1_ = 13.2, l _2_ = 17.0 and l _*i*,max_ = l _*i*_ – 4. The net displacement accrued over one cycle of the magnetic field is Δ*x*_2_ = 0.769. This gives an average speed of *v* = 0.483 over the full cycle or *v* = 22.0 μm s^–1^ in physical units when using the scales described in Section 2.4. (b) Associated driving magnetic field *h*(*t*) of triangular shape with constant slew rate d*h*/d*t* = ± 0.59 between *h*_min_ = 0.55 and *h*_max_ = 1.02, which has then been clipped to the interval [0,1]. The period is thus 1.59. One unit of time on the abscissa corresponds to 2.2 × 10^–2^ s.

If a different driving field is used, which oscillates too rapidly or does not reach a high enough maximum or low enough minimum during one period, the swimmer does not complete an entire cycle of both collapses and detachments, so that it cannot achieve significant net displacement. This dependence can be used to independently control multiple swimmers individually, as shown in Figure 7. There, appropriate adjustment of the maximum and minimum field magnitudes allows to either address one or the other of the two swimmers in a way to induce substantial net motion. In contrast to that, the second swimmer remains basically at rest during that time.

**Figure 7:**
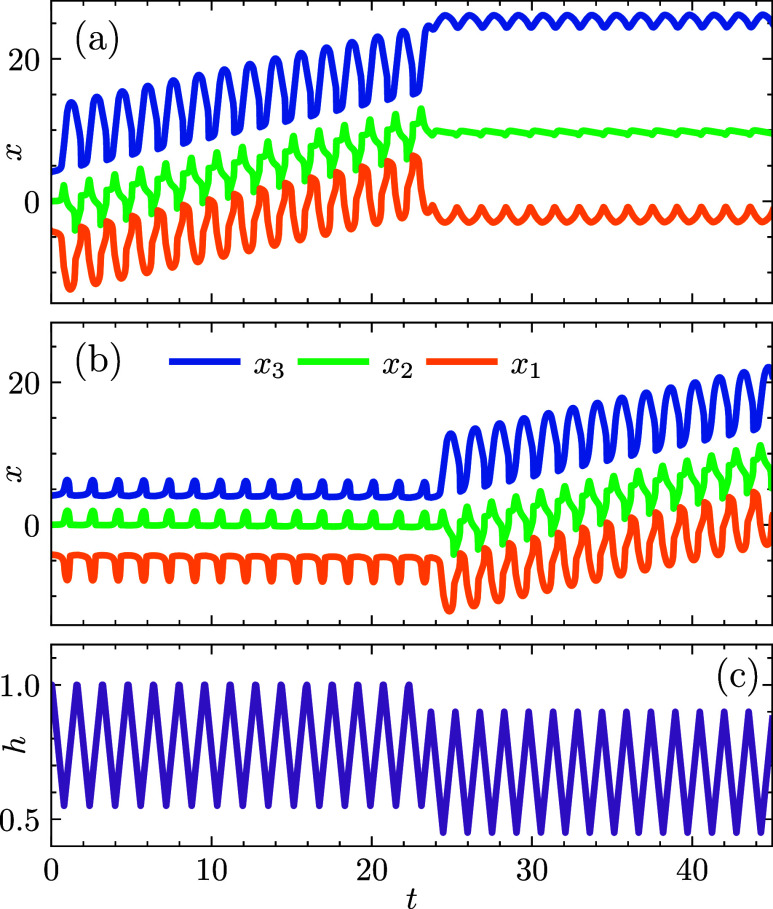
(a) The trajectories of a three-sphere microswimmer with parameters *c*_1_ = 8.1, *c*_2_ = 9.8, l _1_ = 13.2, l _2_ = 17.0 and l _*i*,max_ = l _*i*_ – 4, identically to Figure 6. At first, when the driving magnetic field has the right magnitude, the swimmer completes full cycles leading to net displacement. When the driving field is decreased in magnitude, the swimmer does not reach the fully collapsed state and by lack of pronounced nonreciprocity it oscillates in place. (b) Setting the parameters of a second three-sphere microswimmer differently, here to *c*_1_ = 6.0, *c*_2_ = 6.9, l _1_ = 13.0, l _2_ = 17.0, and l _*i*,max_ = l _*i*_ – 4, can facilitate targeted stimulation of one of the swimmers by adjusting the field magnitudes. First, when the driving magnetic field has a larger magnitude, since the springs are weaker, the swimmer never fully expands, and the cycles remain incomplete. The swimmer oscillates basically in place. When the driving field is decreased in magnitude, the swimmer completes full cycles and therefore achieves substantial net displacement. (c) Throughout, the driving magnetic field *h*(*t*) of triangular shape is of fixed slew rate d*h*/d*t* = ± 0.59. Yet, the magnitude switches at one point. For the first 15 periods, the field oscillates between *h*_min_^(1)^ = 0.55 and *h*_max_^(1)^ = 1.02, clipped to the interval [0,1]. Then, for another 15 periods, it oscillates between *h*_min_^(2)^ = 0.45 and *h*_max_^(2)^ = 0.9. One unit of time on the abscissa corresponds to 2.2 × 10^–2^ s.

## 3 Conclusion and Outlook

We have shown that a simple microswimmer consisting of three magnetizable spheres connected by two springs can achieve net propulsion when driven by an oscillating magnetic field, even at low Reynolds numbers. The key ingredient is the hysteretic collapse and detaching of the spheres due to bifurcations of the equilibrium positions induced by the competition of magnetic attraction and elastic counteraction by the springs. Due to the simple design of the swimmer, a straightforward description of its dynamics is possible by expanding hydrodynamic interactions in terms of the inverse intersphere distances. Using an evolutionary optimization strategy, we have improved the swimmer design and driving magnetic field to achieve high swimming speeds under practical constraints. We demonstrate that a microrobot made out of superparamagnetic spheres of radius 1 μm, connected by two springs with stiffnesses on the order of 1 × 10^–4^ N/m with rest lengths of around 13 and 17 μm could reach swimming speeds of around 20 μm s^–1^ when actuated by an oscillating magnetic field of magnitude 1000 Oe and frequencies of approximately 25 Hz.

Artificial bacteria flagella (ABF),^10^ a promising alternative design for a magnetically actuated microswimmer, can achieve similar speeds (18 μm s^–1^) at a comparable scale (16 μm length and 5 μm radius helix).^35^ However, they require a different kind of magnetic actuation, that is, a rotating magnetic field, in ref 10 with an amplitude of 9 mT. Newer designs using hard magnetic materials can reach speeds on the order of millimeters per second^36^ due to higher frequencies of rotating fields. Differently shaped ABFs can be addressed independently by tuning the actuation frequency,^37^ while we in our case switch the field magnitudes to selectively actuate our different swimmers. Our approach uses an order of magnitude stronger magnetic fields but of a simpler geometry, oscillating in magnitude with constant direction. This could make actuation and control more accessible. However, the fabrication of such a three-sphere swimmer may prove to be more challenging than that of artificial bacterial flagella because of the moving parts and the need for appropriate elastic springs.

The proposed design could be realized using superparamagnetic iron oxide,^38^ DNA springs,^39,40^ or 3D printed elastomers^41^ as springs. To produce the strong and rapidly oscillating uniform driving field, a set of liquid-cooled high-performance Maxwell coils could be employed. Alternatively, the magnitude of the driving field can be scaled down together with the spring stiffnesses at the cost of a slower driving frequency and in turn lower swimming speeds. Our employed optimization strategy and some expected adjustability of the magnitudes and frequencies of the external magnetic field can help to mitigate deviations during experimental realization from the calculated theoretical design parameters. Evolutions of this design, which go beyond the simple minimalist model presented here, could include a microrobot that is entirely printed from an elastomer with embedded magnetic particles that deform the robot body under the influence of an external magnetic field. More complex motion patterns, such as beating cilia or undulating sperm tails, could be achievable. We hope that this theoretical and numerical study can inspire future experimental realizations and investigations of such microswimmers.

## 4 Methods

The equations of motion, eq 5, can be numerically integrated using the implicit Kvaerno5 solver^42^ with adaptive time stepping^43^ and absolute tolerance 1 × 10^–6^. An implicit solver is crucial because of the stiffness of the problem. Benefiting from just-in-time compilation and hardware acceleration, the program was implemented using the JAX ecosystem^44^ in the programming language Python. Notably, we used the libraries Diffrax,^45^ Equinox,^46^ and Evosax.^47^ We used the numerical integration of eq 5 to provide the net displacement per cycle in Figure 4a and the loops in the configuration space presented in Figure 4b.

## Supplementary Material



## Data Availability

All the code and data to reproduce the figures are available at https://github.com/TheoLequy/magspheres.

## Notes

The Supporting Information in part A details the analytical derivation of the positions of bifurcation shown in Figure 3. Second, in part B, it estimates the magnitude of the spring stiffnesses for the three-sphere swimmer to exploit the available magnitude of the external magnetic field. And third, in part C, it lists the sixth-order expressions of the mobility matrix introduced in eq (5) (PDF)
