# Supplementary material for: Magnetically Driven Elastic Microswimmers: Exploiting Hysteretic Collapse for Autonomous Propulsion and Independent Control
Source: ACS Nanosci Au. 2026 Mar 25;6(3):482–90. doi: 10.1021/acsnanoscienceau.6c00014 (PMC13281009; doi:10.1021/acsnanoscienceau.6c00014)
Supplement: Supplementary file 1 [file ng6c00014_si_001.pdf]

Supporting Information
Magnetically Driven Elastic Microswimmers: Exploiting Hysteretic Collapse for Autonomous Propulsion and Independent Control

Theo Lequy[1,*] and Andreas M. Menzel[2,*]

[1] *Eidgenössische Technische Hochschule Zürich, Rämistrasse 101, 8092 Zürich, Switzerland*

[2] *Institut für Physik, Otto-von-Guericke-Universität Magdeburg, Universitätsplatz 2, 39106 Magdeburg, Germany*

(Dated: March 19, 2026)

In this supporting information, we first detail the analytical derivation of the positions of bifurcation shown in Fig. 3. Second, we estimate the magnitude of the spring stiffnesses for the three-sphere swimmer to exploit the available magnitude of the external magnetic field. And third, we include the sixth-order expressions of the mobility matrix introduced in Eq. (5).

A. Analytical expressions for bifurcation locations

The equilibrium positions of the two-sphere setup are found from $\partial U_2/\partial r = 0$, see Eq. (3). From there, we obtain a quintic polynomial equation in r,

$$0 = r^5 - r^4 + \frac{\beta^2}{\epsilon^2}\left(\epsilon^2 - (r-1)^2\right) . \qquad \text{(S1)}$$

However, among the resulting roots, at most three are physical with $r \in (1-\epsilon, 1+\epsilon)$.

Saddle-node bifurcations occur at the local extrema of β. Setting $\mathrm{d}\beta/\mathrm{d}r = 0$, we obtain a cubic polynomial equation,

$$0 = 3r^3 - 10r^2 + (11 - 5\epsilon^2)r + 4(\epsilon^2 - 1)\,, \qquad \text{(S2)}$$

with discriminant

$$\Delta = 4\epsilon^2(3\epsilon^2 - 1)(125\epsilon^2 + 1)\,. \qquad \text{(S3)}$$

Only for $\epsilon \in (1/\sqrt{3}, 1]$ the discriminant is positive and two physical bifurcations occur. At $\epsilon = 1/\sqrt{3}$, both bifurcations merge at $r = 2/3$ and $\beta = 2\sqrt{2}/9$.

B. Magnitude of the spring stiffnesses of the three-sphere swimmer

As can be inferred from Fig. 3(b) of the main article, the strength of the magnetic field for collapse (blue dash-dotted line) does not significantly vary with extensibility ϵ, so that we can approximate

$$\beta_{\text{collapse}} \sim 0.3\,. \qquad \text{(S4)}$$

We use this result from the two-sphere dynamics, which describes the basic underlying hysteretic effect, for our following estimate for the three-sphere case. That is, we only consider the two spheres $(i,j) = (1,2)$ or $(2,3)$ and ignore the weaker influence of the third sphere, which is significantly further away from the outer sphere than the central one. To transform from the rescaled dimensionless strength of the magnetic field β in Sec. II A to the magnetic field parameter h, we use the definition of β introduced before Eq. (3) in the main text. For each of the two mentioned pairs of spheres (i,j), this definition for the rescaled magnetic field at collapse can be rewritten as

$$\begin{aligned}\beta_{\text{collapse},ij} &= H_{\text{collapse},ij}\sqrt{8\pi\chi_a^2\mu_0 a^6/3k_i\ell_i^5} \\ &= \frac{H_{\text{collapse},ij}}{H_{\max}}\sqrt{\frac{8\pi\chi_a^2\mu_0 a H_{\max}^2}{3k_i}\frac{a^5}{\ell_i^5}}\,. \end{aligned} \qquad \text{(S5)}$$

In this expression, we recognize our rescaled quantities as introduced before Eq. (4) in the main text. Spring constants k_i are measured in units of $8\pi\chi_a^2\mu_0 a H_{\max}^2/3$, rest lengths ℓ_i in units of a, and the magnetic field in units of $H_{\max}$, where we defined $h = H/H_{\max}$.

Together, we infer from Eq. (S5) in rescaled units the relation

$$h_{\text{collapse},ij} = \beta_{\text{collapse},ij}\sqrt{k_i\ell_i^5}\,. \qquad \text{(S6)}$$

In combination with Eq. (S4) and in terms of the stiffness parameters $c_i = k_i\ell_i^5$ introduced in Sec. II B, we therefore obtain

$$h_{\text{collapse},ij} \sim 0.3\sqrt{c_i}\,. \qquad \text{(S7)}$$

Thus, if we require $h_{\text{collapse},ij} \sim 1$, we find $c_i \sim 10$.

C. Mobility matrix

In Ref. 1, expressions for the displaceability matrix for spherical particles in a linearly elastic medium were derived up to the sixth order in the inverse particle separation distance. These results become equivalent to those for Stokes flow when we take the limit of $\nu \to 0.5$ for the Poisson ratio. Utilizing the one-dimensionality of our setup, every entry in the mobility matrix becomes a

* tlequy@ethz.ch
* a.menzel@ovgu.de

scalar. Then, the diagonal and off-diagonal entries read

$$M_{ii} = 1 + \sum_{k\neq i} \left[-3.75 x_{ik}^{-4} + 5.5 x_{ik}^{-6}\right], \tag{S8a}$$

$$M_{ij,i\neq j} = 1.5|x_{ij}|^{-3} + \Big[-3.75\sigma_{ij} x_{ik}^{-2} x_{jk}^{-2} + 6\sigma_{ij}\left(x_{ik}^{-2} x_{jk}^{-4} + x_{ik}^{-4} x_{jk}^{-2}\right) - 6.5|x_{ik}^{-3} x_{jk}^{-3}|\Big]_{k\neq i,j}, \tag{S8b}$$

respectively. The sign of some terms is flipped if the kth sphere is located in between spheres i and j, according to

$$\sigma_{ij} = \begin{cases} -1, & \text{if } \{i,j\} = \{1,3\}, \\ +1, & \text{otherwise}. \end{cases} \tag{S9}$$

Since, in the main text, we limit the maximum approach of two spheres to a surface-to-surface distance of one diameter of the spheres, truncation beyond the sixth order leads to only little quantitative deviations.

[1] M. Puljiz and A. M. Menzel, Displacement field around a rigid sphere in a compressible elastic environment, corresponding higher-order Faxén relations, as well as higher-order displaceability and rotateability matrices, Physical Review E **99**, 053002 (2019).
